# Isolated heart model demonstrates evidence of contractile and diastolic dysfunction in right ventricles from rats with sugen/hypoxia‐induced pulmonary hypertension

**DOI:** 10.14814/phy2.13438

**Published:** 2017-10-16

**Authors:** Evandro M. Neto‐Neves, Andrea L. Frump, Alexandra Vayl, Jeffrey A. Kline, Tim Lahm

**Affiliations:** ^1^ Department of Emergency Medicine Indiana University School of Medicine Indianapolis Indiana; ^2^ Department of Medicine Division of Pulmonary Critical Care, Occupational and Sleep Medicine Indiana University School of Medicine Indianapolis Indiana; ^3^ Department of Cellular and Integrative Physiology Indiana University School of Medicine Indianapolis Indiana; ^4^ Richard L. Roudebush Veterans Affairs Medical Center Indianapolis Indiana; ^5^ Present address: Department of Pharmacology Ribeirao Preto Medical School University of Sao Paulo Ribeirao Preto Sao Paulo Brazil

**Keywords:** apelin, apoptosis, bcl‐2/bax, Langendorff, p38MAPK, right ventricular hypertrophy

## Abstract

Although extensively used for the study of left ventricular function, limited experience exists with the isolated heart model in the evaluation of right ventricular (RV) function. In particular, no published experience exists with this tool in sugen/hypoxia‐induced pulmonary hypertension (SuHx‐PH), a frequently used model of severe and progressive PH. We sought to characterize markers of RV contractile and diastolic function in SuHx‐PH and to establish their relationship with markers of maladaptive RV remodeling. Hearts were excised from anesthetized Sprague Dawley rats with or without SuHx‐PH and perfused via the aorta using a Langendorff preparation. We explored the Frank–Starling relationship of RV function (RV developed pressure, d*P*/d*t*_max_, and d*P*/d*t*_min_; all normalized to RV mass) by increasing RV end‐diastolic pressure (RVEDP) from 0 to 40 mmHg. Functional studies were complemented by quantification of RV pro‐apoptotic signaling (bcl2/bax), procontractile signaling (apelin), and stress response signaling (p38MAPK activation). Pearson's correlation analysis was performed for functional and biochemical parameters. SuHx‐RVs exhibited severe RV dysfunction with marked hypertrophy and decreased echocardiographic cardiac output. For any given RVEDP, SuHx‐RVs demonstrated less developed pressure and lower d*P*/d*t*_max_, as well as less pronounced d*P*/d*t*_min_, suggestive of decreased contractile and diastolic function. SuHx‐RVs exhibited decreased bcl2/bax ratios, apelin expression, and p38MAPK activation. Bcl2/bax and apelin RNA abundance correlated positively with RV developed pressure and d*P*/d*t*_max_ and negatively with d*P*/d*t*_min_. p38MAPK activation correlated positively with RV developed pressure. We conclude that SuHx‐RVs exhibit severe contractile and diastolic dysfunction. Increased pro‐apoptotic signaling and attenuated procontractile and stress response signaling may contribute to these functional alterations.

## Introduction

Pulmonary arterial hypertension (PAH) is a progressive and devastating disease characterized by muscularization, dysregulated vasoconstriction, and formation of occlusive lesions in the pulmonary arteries (Rabinovitch 2012; Tuder et al. 2013a,b). These changes lead to increased right ventricular (RV) afterload and, if left untreated, RV failure and death (Rabinovitch 2012; Tuder et al. 2013a,b). The RV initially compensates for the increase in afterload through hypertrophy and increased contractile function. However, as PAH continues to worsen, the RV's compensatory mechanisms fail, resulting in contractile and diastolic dysfunction (Rain et al. 2013; Hsu et al. 2016). These processes ultimately lead to RV failure and eventually death (Vonk‐Noordegraaf et al. 2013; Vonk Noordegraaf et al. 2017). Consequentially, in patients with PAH, decreased RV function is one of the strongest predictors of death (Humbert et al. 2010; Van De Veerdonk et al. 2011). Despite the critical importance of RV function to outcomes in PAH, no RV‐directed therapies exist. While the RV and left ventricle (LV) share several similarities, they are embryologically, structurally, physiologically, biochemically, and electrophysiologically distinct from each other (Friedberg and Redington 2014). It is therefore not surprising that treatment strategies beneficial in LV failure may be detrimental in RV failure (Andersen et al. 2014; Van Campen et al. 2016). This emphasizes that paradigms in LV research cannot be extrapolated to the RV and that specific studies of the RV are necessary. This has recently been identified as a priority by the NIH and the American Thoracic Society (Voelkel et al. 2006; Erzurum et al. 2010; Dweik et al. 2014).

In order to develop RV‐targeted therapeutic strategies, a detailed understanding of their effects on RV function is necessary. While several treatment strategies have been shown to improve RV function in PAH, many of these interventions concomitantly target the pulmonary vasculature, making it challenging to differentiate their pulmonary vascular effects from their potential effects on intrinsic RV function (Vonk‐Noordegraaf et al. 2013, 2017). In order to circumvent this problem, isolated heart models have been used (Nagendran et al. 2007, 2013; Handoko et al. 2009; Piao et al. 2010; Umar et al. 2012; Temple et al. 2016). These models allow for the detailed study of RV contractile and diastolic function independent of changes in the pulmonary vasculature.

However, all these studies have been performed using the monocrotaline model of PAH, whereas no such studies have been performed in the sugen/hypoxia (SuHx) model of PAH. This is of importance, as the SuHx model currently is widely used for the study of PAH pathogenesis and treatment (Stenmark et al. 2009; Maarman et al. 2013). The SuHx model entails a one‐time administration of the VEGF receptor 2 antagonist Su5416 (sugen), followed by 3–4 weeks of hypoxia exposure and re‐exposure to room air for 4 or more weeks (Taraseviciene‐Stewart et al. 2001). This results in severe, angioproliferative PH that mimics human PAH. The model is also characterized by marked RV dysfunction and a progressive course, both of which are important features of human PAH. Because of the widespread use of the SuHx model, there is a need to better characterize the purported contractile and diastolic function of the SuHx‐RV in the isolated heart model. As many in vivo markers of RV function are preload‐dependent, such ex vivo studies would also allow for evaluation of RV function in a setting that is independent of preload. A better understanding of the SuHx‐RV's contractile and diastolic properties and their relation to pathophysiologically relevant molecular signaling pathways will (1) provide a better insight into mechanisms of RV adaptation in this model and (2) facilitate the development of RV‐specific therapies for patients with PAH and other types of PH.

We aimed to characterize the contractile and diastolic function of isolated SuHx‐RVs in a Langendorff model and establish their correlations with pro‐apoptotic signaling (bcl‐2/bax), procontractile signaling (apelin), and activation of the stress response regulator p38MAPK. We report a decrease in RV contractile and diastolic function in SuHx‐RVs and demonstrate novel correlations between changes in RV contractile and diastolic function and alterations in bcl‐2/bax and apelin expression as well as p38MAPK activation.

## Methods

### Animals

Studies were performed in adult male Sprague Dawley rats (175–200 g; Charles River, Wilmington, MA). Animals were allowed ad libitum access to food and water for the duration of the experimentation. All animals received care in compliance with the Guide for the Care and Use of Laboratory Animals and followed the Declaration of Helsinki conventions for the use and care of animals. All experiments were approved by the Institutional Animal Care and Use Committee of the Indiana University School of Medicine.

### Induction of SuHx‐PH

Animals received Su5416 (20 mg/kg subcutaneously; dissolved in DMSO; Sigma‐Aldrich, St Louis, MO) followed immediately by hypoxia exposure. Hypoxia exposure occurred in a hypobaric chamber (*P*_atm_ = 362 mmHg; equivalent to 10% FiO_2_) as described previously (Lahm et al. 2012). After 3 weeks of hypoxia, animals were returned to room air for another 4 weeks. Age‐matched animals not receiving Su5416 and not undergoing hypoxia exposure were employed as control (Sham) groups. Echocardiographic and hemodynamic endpoints in SuHx and control rats from our laboratory have previously been described in detail (Frump et al. 2015; Lahm et al. 2016).

### RV function evaluation by echocardiography

Myocardial structure and function were assessed by echocardiography using a high‐resolution ultrasound system (Vevo 2100, Visual Sonics, Toronto, Canada). Rats were lightly anesthetized with 2% isoflurane via nose cone. Images were obtained in the parasternal two‐dimensional short axis and long axis with an 13‐ to 24‐MHz scan probe. Short‐axis M mode was utilized to measure RV wall thickness. Long‐axis images were used to measure the right ventricular outflow tract (RVOT) at the level of the pulmonary valve. Stroke volume (SV) and cardiac output (CO) were determined using standard formula as follows: SV = VTI × 3.142 × (1/ 2 RVOT) and CO = SV × heart rate (HR) as described previously (Frump et al. 2015; Lahm et al. 2016).

### Ex vivo isolated perfused heart preparation

**Figure 1:**
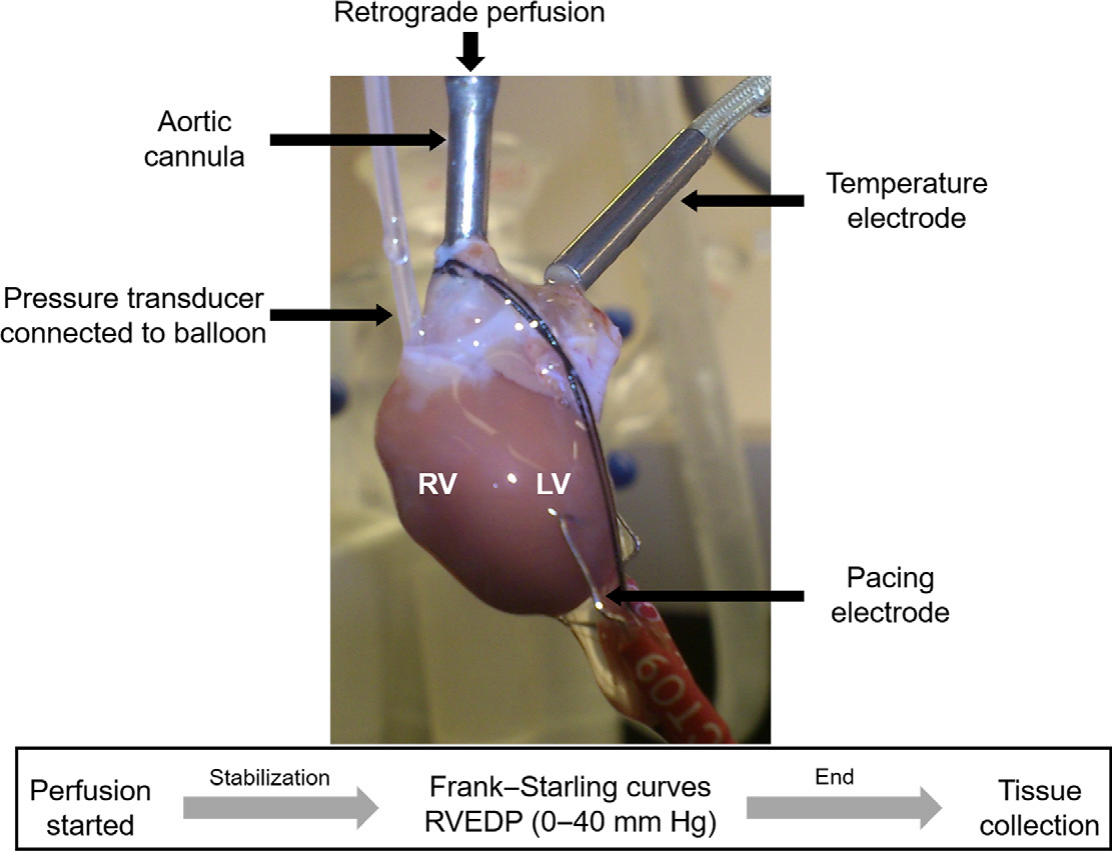
Overview of Langendorff preparation (isolated perfused heart) used to evaluate RV function ex vivo. The representative picture shows a rat heart perfused with oxygenated Krebs–Henseleit buffer delivered in a retrograde direction down the aorta at a constant pressure (60 mmHg). A fluid‐filled balloon connected to a pressure transducer is positioned into the RV to acquire parameters of RV function at increasing RV end‐diastolic pressures (RVEDP; 0–40 mmHg). The temperature electrode is used to maintain a constant temperature. The pacing electrode is used to generate a constant heart rate.

To perform the heart perfusion model, hearts were rapidly isolated and perfused via the aorta at a constant pressure (60 mmHg) with oxygenated Krebs–Henseleit bicarbonate buffer (95% O_2_ + 5% CO_2)_, in a temperature‐controlled (37°C) Langendorff apparatus as previously described (Watts et al. 2006; Zagorski et al. 2007). Electrodes were placed at the aorta cannula and cardiac apex, and hearts were paced at 350 beats/minute (Grass Instruments, S44 stimulator). Parameters of RV systolic and diastolic function (developed pressure, maximum rate of pressure development [d*P*/d*t*_max_], and maximum rate of relaxation [d*P*/d*t*_min_]) were measured through a high‐compliance fluid‐filled balloon with a diameter of 3 mm inserted into the RV and connected to a pressure transducer. Each balloon was pretested for filling capacity before the beginning of the experiments. Parameters were recorded using a PowerLab data acquisition system (AD Instruments, Australia), and transducer calibration was performed before each experiment following the manufacturer's instructions. Frank–Starling relationship was achieved in each heart while filling the balloon with saline until RV end‐diastolic pressure (RVEDP) increased in 5 mmHg increments from 0 to 40 mmHg. The experimental setup is depicted in Figure 1. After ex vivo perfusion, RVs were carefully separated from the left ventricle and septum (LV+S). Fulton index (RV mass divided by mass of LV plus septum; RV/[LV + S]) was calculated for determination of RV hypertrophy.

### Western blot analysis

Immediately after ex vivo analysis, RV tissue was then washed in ice‐cold saline and snap‐frozen. RV tissue was then homogenized using an Omni international tissue grinder (Thermo Fisher Scientific, Waltham, MA) in ice‐cold RIPA lysis buffer (Thermo Fisher) containing proteinase inhibitor cocktail (EMD Millipore/Sigma‐Aldrich, St. Louis, MO) and PhosStop inhibitor cocktail (Roche, Indianapolis, IN). After homogenization, lysate was sonicated for ten one‐second pulses at 100% power and then centrifuged. The supernatant was saved and used as whole lung lysate. Protein concentration was measured using BCA Protein Assay (Thermo Fisher). Rabbit polyclonal anti‐phospho‐p38MAPK, anti‐total p38MAPK, anti‐bcl2, and anti‐bax (all used at 1:1000, and from Cell Signaling, Danvers, MA) and mouse monoclonal anti‐Vinculin loading control (1:5000; Calbiochem; Billerica, MA) primary antibodies were used, all diluted in Pierce Protein‐Free T20 Blocking Buffer (Thermo Fisher). All antibodies used have been extensively validated (Lin et al. 2005; Bernal‐Mizrachi et al. 2006; Bikkavilli et al. 2008; Tang et al. 2008; Slone et al. 2011; Choi et al. 2016; Jiang et al. 2016; Zhang et al. 2017). Rabbit‐HRP (Cell Signaling) and mouse‐HRP (KPL, Gaithersburg, MD) secondary antibodies were diluted 1:2000 in Pierce Protein‐Free T20 Blocking Buffer (Thermo Fisher). Densitometry was performed using ImageJ.

### Quantitative real‐time PCR

Total RNA from 10 to 30 mg snap‐frozen RV tissue was extracted using the RNeasy Fibrous Tissue kit (Qiagen, Germantown, MD) and quantified with a NanoDrop 2000 Spectrophotometer (Thermo Scientific, Wilmington, DE). To generate cDNA for real‐time PCR analysis, 1 *μ*g total RNA was reverse‐transcribed using the SuperScript III First‐Strand Synthesis System for RT‐PCR (Life Technologies, Thermo Fisher, Carlsbad, CA). Real‐time PCRs were performed using the Applied Biosystems 7500 Real‐time PCR system (Applied Biosystems, Thermo Fisher, Foster City, CA). TaqMan gene expression assays for *Apln* (assay ID: Rn00581093_m1; Thermo Fisher) and *Hprt1* (endogenous control; assay ID: Rn01527840_m1; Thermo Fisher) were used. Changes in mRNA expression were determined by the comparative CT (2^−∆∆CT^) method. Data were expressed as fold change to normoxic controls.

### Statistical analysis

Data are presented as means ± SEM. Echocardiography, Fulton index, and biochemical results were compared by Student's *t*‐test. Langendorff data were compared with two‐way ANOVA followed by Bonferroni post hoc test. Correlation analyses were performed by determining Pearson's correlation coefficient. The null hypothesis was rejected at *P* < 0.05. All tests were performed using GraphPad Prism software (version 5.0, La Jolla, CA).

## Results

### SuHx rats exhibit RV hypertrophy and decreased RV cardiac output in vivo

**Figure 2:**
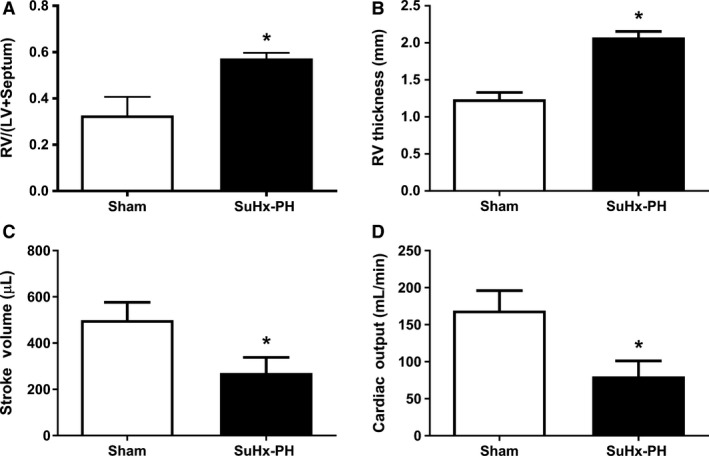
SuHx‐PH induces RV hypertrophy and decreases RV stroke volume and cardiac output. Fulton index (A) and echocardiographic parameters of RV remodeling (RV free wall thickness; (B) and function (stroke volume and cardiac output; C and D) in Sham and SuHx‐PH rats. *N* = 6/group. Values are means ± SEM. *P <* *0.05 by unpaired *t*‐test.

We first characterized our experimental animals by measuring parameters of RV hypertrophy (RV/[LV + S] ratio by Fulton index and RV wall thickness by echocardiography) and RV function (stroke volume and cardiac output by echocardiography). As expected, we noted robust increases in RV hypertrophy in SuHx rats (Fig. 2A and B), as well as 50–60% decreases in RV stroke volume (Fig. 2C) and cardiac output (Fig. 2D).

### SuHx‐RVs exhibit decreased contractile and diastolic function

**Figure 3:**
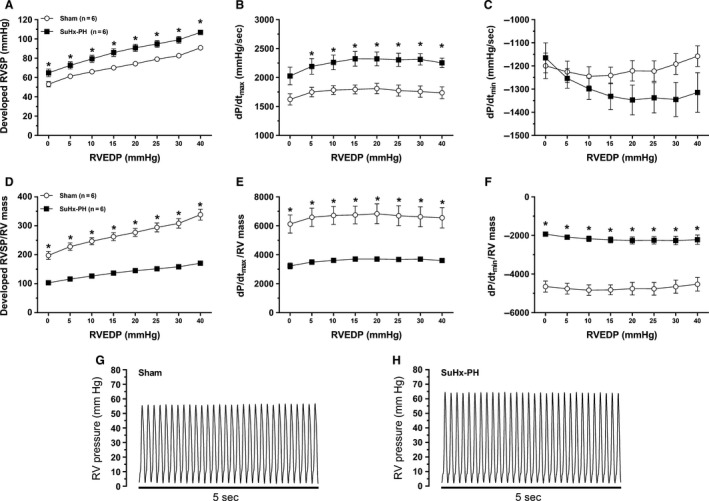
Isolated and perfused right ventricles from SuHx rats exhibit systolic and diastolic impairment. Isolated perfused heart model was used to evaluate parameters of intrinsic RV function (expressed as developed RV systolic pressure [RVSP], d*P*/d*t*_max_, and d*P*/d*t*_min_ for various degrees of RV end‐diastolic pressure [RVEDP]). Absolute values are shown in panels (A) (RVSP), (B) (d*P*/d*t*_max_), and (C) (d*P*/d*t*_min_). Developed RVSP, d*P*/d*t*_max_, and d*P*/d*t*_min_ normalized for RV mass (used as a measure of abundance of contractile units) are shown in panels (D), (E), and (F), respectively. Note that RV dysfunction (systolic and diastolic) for SuHx‐PH is only observed when values are normalized for RV mass. Values are means±SEM. *P < 0.05 SuHx‐PH versus Sham (two‐way ANOVA). (G) and (H) show representative tracings for Sham and SuHx‐PH groups, respectively.

We next evaluated the intrinsic function of isolated SuHx‐RVs using the Langendorff preparation. When measuring developed RVSP, d*P*/d*t*_max_, and d*P*/d*t*_min_ over a wide range of RV filling pressures (RVEDP 0–40 mmHg) and comparing values to control rats, SuHx‐RVs exhibited modest increases in developed RVSP (for RVEDP between 15 and 40 mmHg; Fig. 3A) and d*P*/d*t*_max_ (RVEDP 10–40 mmHg; Fig. 3B), and no significant changes in d*P*/d*t*_min_ (Fig. 3C). However, when normalizing data for RV mass (reflecting the abundance in contractile units), we observed 50% impairments in developed RVSP (Fig. 3D), d*P*/d*t*_max_ (Fig. 3E), and d*P*/d*t*_min_ (Fig. 3F) in SuHx‐RVs. These alterations were noted over the entire RVEDP range, indicating profound impairments in RV contractile and diastolic function in SuHx‐RVs.

### SuHx‐RVs exhibit increased pro‐apoptotic signaling, decreased apelin mRNA expression, and decreased p38MAPK activation

**Figure 4:**
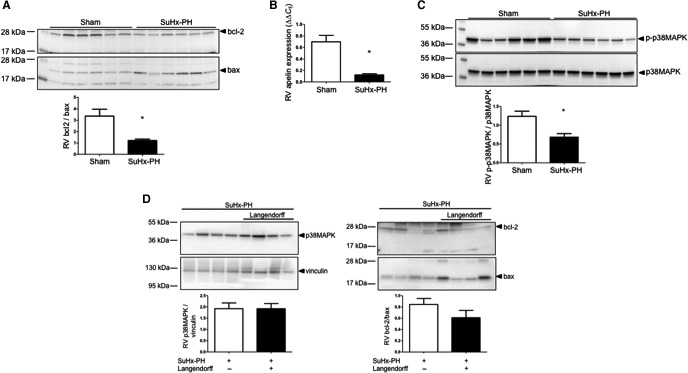
RVs from SuHx‐PH rats exhibit decreased bcl‐2/bax ratio, apelin expression, and p38MAPK activation. (A) Western blot analysis of bcl‐2/bax ratio (ratio of prosurvival [bcl‐2] to pro‐apoptotic [bax] signaling) in RV homogenates measured by Western blot and densitometric analysis (below Western blot). (B) RV apelin mRNA expression in RVs measured by quantitative real‐time RT‐PCR. (C) RV expression of phospho‐p38MAPK normalized to total p38MAPK analyzed by Western blot with densitometry below. (D) Comparison of p38MAPK abundance and bcl‐2/bax between SuHx‐RVs that underwent ex vivo testing in the Langendorff apparatus (black bars) and freshly isolated SuHx‐RVs that did not undergo ex vivo testing (white bars). *N* = 6/group in (A–C), *n* = 4/group in (D). Values are means ± SEM. *P <* *0.05 by unpaired *t*‐test.

In order to study biochemical alterations that may underlie the observed functional impairment, we measured bcl‐2/bax ratio (as a marker of pro‐apoptotic signaling), apelin expression (as a marker of procontractile signaling), and p38MAPK activation (as a marker of stress response signaling). As previously demonstrated, we noted a decrease in bcl‐2/bax (Fig. 4A) as well as apelin mRNA (Fig. 4B), suggestive of increased pro‐apoptotic signaling and decreased procontractile signaling. In addition, we noted a decreased in p38MAPK activation (Fig.**** 4C), suggesting decreased stress signaling. These decreases were very robust, ranging from 50% for bcl2/bax to 90% for apelin.

To rule out confounding effects of the ex vivo preparation on the biochemical endpoints studied, we compared p38MAPK abundance and bcl‐2/bax ratios between SuHx‐RVs that underwent ex vivo testing in the Langendorff apparatus and freshly isolated SuHx‐RVs that did not undergo ex vivo testing. Of note, while the bcl‐2/bax ratio tended to be slightly decreased in Langendorff vs. non‐Langendorff RVs, we did not find any significant differences in these parameters between Langendorff and non‐Langendorff RVs (Fig. 4D), suggesting that the changes noted are due to PH rather than the ex vivo preparation.

### Markers of RV contractile and diastolic function correlate with pro‐apoptotic, procontractile, and stress response signaling parameters

**Figure 5:**
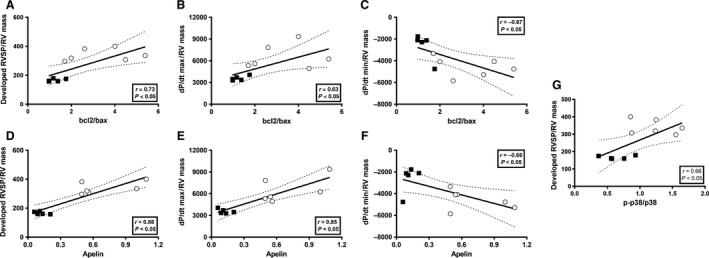
Langendorff‐derived parameters of RV function correlate with markers of pro‐apoptotic signaling, procontractile signaling, and stress response signaling in Sham and SuHx‐PH rats. Developed RVSP, d*P*/d*t* max, and d*P*/d*t*_min_ (all at 40 mmHg of RVEDP) were correlated with RV bcl2/bax ratio (A–C), apelin (D–F), and p38MAPK activation (G) using Pearson's correlation analysis. Each dot represents one data pair (Sham represented by open circles, SuHx‐PH represented by black squares).

In order to investigate relationships between RV function parameters and markers of pro‐apoptotic, procontractile, and stress response signaling, we correlated developed RVSP, d*P*/d*t*_max_, and d*P*/d*t*_min_ (all at 40 mmHg of RVEDP and normalized for RV mass) with bcl‐2/bax, apelin mRNA, and p38MAPK activation. We noted moderate‐to‐strong correlations between developed RVSP and bcl‐2/bax (Fig. 5A) and d*P*/d*t*_max_ and bcl‐2/bax (Fig. 5B), as well as a strong negative correlation between d*P*/d*t*_min_ and bcl‐2/bax (Fig. 5C), indicating more preserved RV contractile and diastolic function with less pronounced pro‐apoptotic signaling. Similarly, we noted moderate‐to‐strong correlations between developed RVSP and apelin (Fig. 5D) and d*P*/d*t*_max_ and apelin (Fig. 5E), as well as a strong negative correlation between d*P*/d*t*_min_ and apelin (Fig.**** 5F), suggesting more preserved RV contractile and diastolic function with more preserved apelin expression. Interestingly, the positive correlations between apelin and developed RVSP as well as d*P*/d*t*_max_ were the most pronounced among all correlations studied, suggesting a particular tight connection between apelin expression and RV contractile function. p38MAPK activation correlated with developed RVSP (Fig. 5G), but not d*P*/d*t*_max_ or d*P*/d*t*_min_. Taken together, these correlations suggest potentially clinically relevant relationships between RV function parameters and markers of pro‐apoptotic, procontractile, and stress response signaling.

## Discussion

Our study is the first to characterize the SuHx‐RV in an isolated heart model. We demonstrate robust decreases in RV contractile and diastolic function. The correlations of RV contractile and diastolic function endpoints with markers of pro‐apoptotic signaling, procontractile signaling, and stress response signaling suggest that alterations in these pathways contribute to RV contractile and diastolic dysfunction.

The Langendorff technique of isolated heart perfusion has long been used for studies of LV function in models of acute or chronic LV injury (Bell et al. 2011). Modification of the preparation for studies of the RV is technically more challenging, and the Langendorff technique has only rarely been used for studies of RV function. Published studies used the preparation in order to study RV contractile or electrophysiological function (Nagendran et al. 2007, 2013; Handoko et al. 2009; Piao et al. 2010; Umar et al. 2012; Temple et al. 2016). Of note, all these studies have been performed in the monocrotaline model of PAH, a model which only incompletely recapitulates the human PAH phenotype (Stenmark et al. 2009; Maarman et al. 2013). In addition, these studies did not evaluate RV diastolic function, a clinically highly relevant parameter (Rain et al. 2013). We have recently employed this preparation for characterization of a novel rat model of chronic thromboembolic PH (CTEPH) (Neto‐Neves et al. 2017). However, to the best of our knowledge, the Langendorff preparation has not yet been employed for the study of RVs from SuHx rats. This is a significant knowledge gap, as this currently is one of the most widely used animal models of PH. It is therefore important to thoroughly characterize the features of RV function and dysfunction in this model.

Comparison of our results with those performed in monocrotaline PH is difficult, as these studies focused on before‐and‐after interventions rather than describing RV contractile function for various degrees of RVEDP. In addition, these studies did not describe diastolic RV function. We recently employed the isolated heart preparation in a novel rat model of CTEPH, where we noted extensive RV contractile dysfunction (also characterized by echocardiography and pressure volume relationship in vivo), similar to what we observed in our current model (Neto‐Neves et al. 2017). However, no diastolic dysfunction was observed in CTEPH RVs. RV contractile dysfunction was also previously observed in RVs from rats with moderately severe PH induced by acute pulmonary embolism (Watts et al. 2006; Zagorski et al. 2007). Of course, these RVs differ from chronic PH RVs, as they do not exhibit RV hypertrophy or other compensatory mechanisms.

Our study advances the field by (1) demonstrating that the Langendorff preparation is feasible in SuHx‐PH, (2) characterizing the contractile and diastolic phenotype of the SuHx‐RV, and (3) identifying novel correlations between physiological markers of RV function and molecular signaling parameters. While RV contractile and diastolic function have previously been characterized in SuHx rats in vivo using pressure–volume loops (Da Silva Gonçalves Bos et al. 2017), our study further advances the field by studying RV function independently of increases in pulmonary vascular resistance and by investigating developed RVSP, d*P*/d*t*_max_, and d*P*/d*t*_min_ over a wide range of RV filling pressures (RVEDP 0–40 mmHg). Our studies also demonstrate the importance of properly normalizing the readouts obtained in this model. As demonstrated in Figure 3, when developed RVSP, d*P*/d*t*_max_, and d*P*/d*t*_min_ are not normalized for RV mass (reflecting the abundance of contractile units), it appears that SuHx‐RVs exhibit better contractile and diastolic function than controls [similar to what was observed when investigating contractile function in isolated monocrotaline RVs (Nagendran et al. 2007, 2013)]. However, SuHx‐RVs exhibit twice the mass than normoxia controls, resulting in less contractile force and relaxation per contractile unit. We believe that this normalization step is important and recommend that markers of contractile and diastolic function are normalized for RV mass.

One particular advantage of ex vivo preparations such as the Langendorff is that the effect of acute interventions on PH‐RVs can be studied under tightly regulated conditions, allowing for a detailed and highly controlled characterization of acute interventions on RV function. For example, drugs, inhibitors, or activators can be added to the perfusate, allowing for the detailed study of time‐ or dose‐dependent effects on RV function endpoints. In addition to performing functional studies, the Langendorff technique can also be used for cardiomyocyte isolation. Isolated cardiomyocytes generally have been understudied in animal models of PH, and more familiarity with the Langendorff preparation may advance this area. We have successfully utilized this technique in SuHx‐PH rats, allowing for mechanistic studies in cultured cardiomyocytes from untreated or treated animals.

Cardiomyocyte apoptosis has been linked to the development of RV failure (Bogaard et al. 2009, 2010). We therefore evaluated the ratio of the apoptosis inhibitor bcl‐2 and the apoptosis mediator bax, with a lower bcl‐2/bax ratio indicating a shift toward pro‐apoptotic signaling (Misao et al. 1996; Condorelli et al. 1999). As reported before (Frump et al. 2015; Lahm et al. 2016), we noted a decreased bcl‐2/bax ratio in SuHx‐RVs. We now report the novel finding that decreases in the bcl‐2/bax ratio correlate with decreases in contractile and diastolic function, suggesting that increased pro‐apoptotic signaling contributes to RV contractile dysfunction and impaired relaxation.

Apelin is a secreted peptide expressed in the vasculature and heart that exerts its effects via the G‐protein‐coupled APJ receptor (Andersen et al. 2011; Dalzell et al. 2015). Apelin plays a critical role in cardiac development, angiogenesis, prosurvival signaling, nitric oxide‐dependent vasodilation, and inotropic signaling (Andersen et al. 2011; Dalzell et al. 2015). In the LV, apelin regulates blood vessel formation after ischemic injury through the upregulation of cell migration (Wang et al. 2013), progenitor cell recruitment and differentiation (Li et al. 2012), tube formation, and regulating vascular integrity (Azizi et al. 2015). Interestingly, hypoxic mice infused with apelin exhibit increased cardiac output (Alastalo et al. 2011), making apelin a promising target for cardiopulmonary diseases. A role for apelin in PH is suggested by studies demonstrating that apelin null mice exhibit worsening PH and that circulating apelin is decreased in PAH patients (Chandra et al. 2011). Emerging data suggest a role of apelin in RV failure. Others and we demonstrated decreases in apelin mRNA and/or protein in RVs from rats with PH‐induced RV failure (Drake et al. 2011; Frump et al. 2015). We detected negative correlations between RV apelin abundance and contractile and diastolic function, suggesting that decreases in apelin contribute to these processes. This is further supported by a recent study, which demonstrated stimulatory effects of apelin and APJ on RV contractile function (Yang et al. 2017). The mechanisms underlying apelin's procontractile effects in the RV are currently under investigation in our laboratory.

The novel finding of a decrease in p38MAPK phosphorylation in the SuHx‐RV at first sight was surprising. p38MAPK is a ubiquitous regulator of inflammatory processes and has been linked to promoting pro‐inflammatory signaling and pro‐inflammatory cytokine activation (Zarubin and Han 2005). We therefore expected to see increased p38MAPK activation in our model, in which we previously demonstrated increased expression of pro‐inflammatory cytokines (Frump et al. 2015). However, p38MAPK effects are highly stimulus‐ and cell type‐dependent. For example, p38MAPK signaling has been shown to promote cell death in some cell lines, but to enhance survival, cell growth, and differentiation in other cell types (Zarubin and Han 2005). Specifically, p38MAPK is a known inducer of the cardioprotective mediators heat‐shock protein 27 and interleukin‐6 (Rane et al. 2003) as well as the cytoprotective mediators heat‐shock protein 70 and carbon monoxide (Kim et al. 2005; Schallner et al. 2011). Furthermore, p38*β* MAPK affords cytoprotection against oxidative stress‐induced astrocyte apoptosis (Shin et al. 2011). A decrease in p38MAPK activation in the pro‐apoptotic environment of the SuHx‐RV is therefore not completely unexpected and is physiologically plausible. Lastly, p38MAPK responses may be time‐dependent and differ between various stages of RV failure development. Future studies will explore these questions in detail.

A theoretical possibility exists that the process of heart isolation causes hypoxia stress that is interacting with the experimental injury and/or confounding by independent effect. However, several lines of evidence suggest this is not the case. First, in order to minimize this possibility, we transferred all hearts from beating in vivo to aortic perfused within a minute and kept them on iced saline until perfused. In addition, all SuHx‐PH hearts were isolated by the same investigator with the same skill and rapidity as the controls. Indeed, our healthy animals showed normal function, suggesting that there is no significant injury occurring during the isolation process. Second, we noted a higher d*P*/d*t*_max_ and lower d*P*/d*t*_min_ in SuHx‐RVs *vs*. controls (Fig. 3B and C; not corrected for RV mass), similar to what was noted by Liu et al. (2017) in SuHx rats in vivo. Lastly, to rule out biochemical differences between SuHx‐RVs that underwent ex vivo testing in the Langendorff apparatus and freshly isolated SuHx‐RVs that did not undergo ex vivo testing, we compared p38MAPK abundance and bcl‐2/bax ratios between the two groups. Of note, we did not find any differences in these parameters between Langendorff and non‐Langendorff RVs (Fig. 4D). While we cannot fully rule out any subtle differences in the parameters studied or differences in other endpoints, taken together, these findings suggest that SuHx‐RVs behave similarly ex vivo than in vivo.”

One difference between the Langendorff preparation using the RV *vs*. the LV is that in case of the RV, the balloon is inserted into a crescent‐shaped rather than a round cavity. We did not perform any ex vivo imaging in order to characterize RV cavity dimensions with the balloon in place, but gross visualization of the RV with the balloon inflated demonstrated a maintained crescent shape of the RV. While we cannot rule out slight changes in RV architecture toward a more spherical form in the area where the balloon is inflated, this still allows squeeze from both circumferential and longitudinal contractile fibers, allowing for assessment of contractile and diastolic function in a more physiological setting than what is assessed with isolated muscle strips. In fact, RV volume and function have been described to correlate well when measured by intracavitary balloon and echocardiography in dogs (Jiang et al. 1994).

Our study has limitations. First, our experimental animals only included males. We previously reported that female SuHx‐PH animals exhibit better RV function than their male counterparts (Frump et al. 2015; Lahm et al. 2016). In order to study animals with the most severe RV failure phenotype, we focused the current study on male animals. Future studies with the Langendorff preparation will focus on female RVs and the comparison of RV function in male vs female animals. Second, while bcl2/bax or apelin correlated robustly with developed RVSP, d*P*/d*t*_max_, and d*P*/d*t*_min_, p38MAPK only correlated with developed RVSP. This could indicate that p38MAPK activation only affects contractile function but does not affect diastolic function. Alternatively, this observation could be a function of more variability in p38MAPK activation versus other parameters, or a result of a type II error. Lastly, our correlations suggest relationships between the studied parameters, but not causation. This will be the focus of future studies.

In summary, this is the first study to demonstrate decreases in RV contractile and diastolic function in isolated SuHx‐RVs using a Langendorff preparation. In addition, we report increased pro‐apoptotic signaling, decreased apelin expression, and decreased p38MAPK activation in SuHx‐RVs. The correlations between markers of RV contractile or diastolic function and markers of pro‐apoptotic signaling, procontractile signaling, and stress response signaling suggest that alterations in these pathways contribute to contractile and diastolic dysfunction in SuHx‐RVs. Future ex vivo studies of SuHx‐RVs may facilitate the development of novel therapeutic options for PAH and other types of PH.

## Conflict of Interest

TL has received consulting fees from Bayer, Actelion, and Gilead. TL's laboratory has received reagents from Eli Lilly & Co.
